# Prior surgical intervention and tumor size impact clinical outcome after precision radiotherapy for the treatment of optic nerve sheath meningiomas (ONSM)

**DOI:** 10.1186/1748-717X-6-117

**Published:** 2011-09-18

**Authors:** Sebastian Adeberg, Thomas Welzel, Stefan Rieken, Jürgen Debus, Stephanie E Combs

**Affiliations:** 1 Department of Radiation Oncology, University Hospital of Heidelberg, Im Neuenheimer Feld 400, 69120 Heidelberg, Germany

**Keywords:** Meningioma, visual outcome, toxicity, local control

## Abstract

**Purpose:**

We analyzed our long-term experience with fractionated stereotactic radiotherapy (FSRT) in patients with meningioma of the optic nerve sheath (ONSM).

**Patients and Methods:**

Between January 1991 and January 2010, 40 patients with ONSM were treated using FSRT. Of these, 19 patients received radiotherapy as primary treatment, and 21 patients were treated after surgical resection. The median target volume was 9.2 ml, median total dose was 54 Gy in median single fractions of 1,8 Gy.

**Results:**

Local progression-free survival was 100%. Median survival after FSRT was 60 months (range 4-228 months). In all patients overall toleration of FSRT was very good. Acute toxicity was mild. Prior to RT, 29 patients complained about any kind of visual impairment including visual field deficits, diplopia or amaurosis. Prior surgical resection was identified as a negative prognostic factor for visual outcome, whereas patients with larger tumor volumes demonstrated a higher number of patients with improvement of pre-existing visual deficits.

**Conclusion:**

Long-term outcome after FSRT for ONSM shows improved vision in patients not treated surgically prior to RT; moreover, the best improvement of visual deficits are observed in patients with larger target volumes. The absence of tumor recurrences supports that FSRT is a strong alternative to surgical resection especially in small tumors without extensive compression of normal tissue structures

## Introduction

Treatment of primary optic nerve sheath meningiomas (ONSM) remains a challenge in the interdisciplinary team of surgeons, opthalmologists and radiation oncologists. They are located directly adjacent to the optic nerve which is sensitive to any treatment damage, including radiation or surgical procedures. They arise from meningothelial cap cells of arachniod villi which surround the optical nerve within the orbit or within the intracanalicular part of the optic nerve.

In general, menigiomas are slow growing tumors with an annual incidence of 6 per 100,000; most patients remain without any clinical symptoms over very long periods of time. ONSM are a rare subtype accounting for 2% of all meningiomas, but they represent the second most frequent optic nerve tumors [1-3]. Initially believed to be extremely rare, ONSM diagnosis increased steadily with continuous optimization of neuroimaging in the CT and MRI era. Benign meningiomas mostly occur in middle-aged or elderly adults, and women are affected twice as often than men. About 30-60% of all intraorbital meningeomas are primary ONSM [3-6]. The vast majority of ONSM are unilateral and become noticeable through painless loss of visual acuity [1,7-12].

For treatment of ONSM, surgical resection can be of choice for certain cases, especially for large tumors leading to intraorbital pressure and compression; for asymptomatic patients, however, also a wait-and-see strategy might be followed, and patients with remaining useful vision and no tumor progression have been observed in the past [13].

One main risk associated with surgery is the often inevitable dissection of the vascular supply of the nerve, which leads to severe visual impairment in about 95% of the patients [3]. There have been only a few series with improved vision after surgical treatment [14,15].

Modern photon radiation techniques such Fractionated Stereotactic Radiotherapy (FSRT) have been established in clinical routine enabling the delivery of highly conformal doses with steep dose gradients to normal tissue. With these techniques, precise treatment of malignant and non-malignant target lesions in close vicinity to organs at risk is possible. For ONSM, several groups have reported excellent clinical outcome with low rates of side effects, however, in small series with only short or mid-term follow-up [4,9-12,16]. In spite of the convincing results in ONSM it is still discussed controversially whether benign ONSM should be irradiated directly after diagnosis, postoperatively after subtotal neurosurgical resection or at the time of clinical or morphological progression during follow-up.

In the present analysis we report our long-term results a large patient group with ONSM treated with FSRT. Special focus is set on the evaluation of prognostic factors as well as long-term preservation of quality of life.

## Patients and Methods

Forty consecutive patients with ONSM treated with FSRT between January 1991 and January 2010 at the Department of Radiation Oncology and the Germany Cancer Research Center (dkfz) in Heidelberg, Germany, were included into this analysis.

Patients were followed prospectively. Additionally, we sent out a detailed questionnaire to all patients asking about recent neurological status including cranial nerve deficits, side effects after treatment during follow-up, additional treatments for ONSM, quality of life prior to and after FSRT as well as any improvement in pre-existing sequelae. This questionnaire was returned in 32 out of the 40 patients (80%).

The median age at the time of radiotherapy was 44 years (range 17-83 years). The tumor manifestation was on the right eye in 16 patients and on the left eye in 23 patients. In one patient both eyes were affected. The female to male ratio was about 2:1 (26 females and 14 males). Patients' characteristics are summarized in table 1.

**Table 1:** Patients' characteristics of 40 patients with ONSM treated with FSRT

Characteristic	No. (%)
**Affected side**	

right	16 (40)

left	23 (58)

bilateral	1 (2)

**Treatment prior to FSRT**	

none	18 (45)

surgery	21 (53)

chemotherapy	1 (2)

**FSRT**	

primary radiotherapy	19 (48)

directly post-surgery	9 (22)

for progression after surgery	12 (30)

**Symptoms**	

Visual field deficits	20 (50)

amaurosis	6 (15)

diplopia	8 (20)

pain	5 (13)

exopthalmia	10 (25)

For 19 patients treatment was recommended as primary treatment, and 21 were treated after surgical resection. Of these, 12 were treated for tumor progression of progressive clinical symptoms during follow up. In 9 patients, RT had been conducted immediately postoperatively. From those who underwent previous surgery all had been initially diagnosed with benign WHO Grade I meningiomas.

The median time between surgical resection and radiation therapy was 43 months.

The median period between surgery and RT was 3 months (range 1-7 months) for those treated immediately postoperatively, and a median of 56 months (range 2-132 months) for those treated for progression of ONSM after surgical resection.

**Figure 1:**
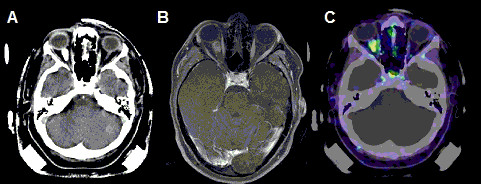
**Treatment-planning examinations for a patient with right-sided ONSM for primary FSRT**. CT (A), MRI (B) and 86-Ga-DOTATOC-Pet (C) were used for treatment planning after image-fusion, a dose of 54 Gy was prescribed in 1.8 Gy single doses.

All patients were treated with FSRT. For treatment planning, patients were fixed with a custom-made Scotch cast^® ^mask; this mask allows an overall positioning accuracy of 1-2 mm. With the mask fixed to the stereotactic base frame, contrast-enhanced CT and MRI scans were performed for treatment planning. For patients without prior histological confirmation, an additional PET using ^68^Ga-DOTATOC was performed as reported previously [17,18] to support the clinical as well as MR-imaging based diagnosis of meningioma. Typical examinations for treatment planning are shown in Figure 1.

We defined the macroscopic lesion visible on contrast-enhanced MRI as the gross tumor volume (GTV), adding 1-2 mm safety margin for the planning target volume (PTV). The median PTV was 9.2 ml. Radiotherapy planning for FSRT was performed using the Voxelplan^® ^software developed at the German Cancer Research Center (dkfz) or the STP software (Stryker, Leibinger). Three to four non-coplanar isocentric fields irregularly shaped with a micro-multileaf collimator were used. The median dose to the PTV was 54 Gy (range 25-66 Gy) in fractions of 1.8 - 5 Gy. None of the patients received concomitant chemotherapy.

All photon plans were delivered using a 6 MeV linear accelerator (Siemens, Erlangen, Germany)

The median follow-up time was 60 months (range from 4 - 228 months). All patients were seen on regular intervals for clinical follow-up, as well as for MR-imaging. The first follow-up examination was scheduled 6 weeks after completion of RT, then in three months intervals for the first year. Thereafter, yearly follow-up visits were scheduled. Clinical examination included thorough neurological assessment and visual assessment through fomal opthalmologis assessment by the opthalmologist. Other examinations, such as endocrinological evaluation, were scheduled depending on the dose distribution of the treatment plan as well as on a clinical basis. Progression-free survival was determined based on the RECIST criteria evaluating two orthogonal diameters of the lesion. Tumor progression was determined by an increase in tumor size of more than 35% (product of the two orthogonal diameters) or any increase in tumor size on subsequent imaging examinations. Overall survival was calculated from the date of the first diagnosis to the last follow-up or death (by any cause). Survival after irradiation was calculated from initiation of irradiation. Progression-free survival was calculated from the first day of irradiation until tumor progression or death (also any cause), whichever occurred first, using the Kaplan-Meier method. Influence on prognostic factors on outcome was assessed using the univariate Cox proportional regression model. Statistical analyses were performed with the software program Statistica 6.1 (StatSoft, Hamburg, Germany).

## Results

### Local control and Survival after FSRT

During follow-up, no patient developed imaging-defined progression of the ONSM after FSRT. Therefore, local progression-free survival was 100% at a median follow-up time of 60 months (range 4-228 months).

Survival was 93% at 5 years after FSRT, all deaths were non-related to the ONSM.

### Overall Toxicity

In all patients overall toleration of FSRT was very good. Acute toxicity was mild. Most patients experienced local alopecia restricted to small regions. Fatigue was a common complaint of one fifth of the 40 patients. Two patients presented with xerophthalmia, and on patient developed acute conjunctivitis during treatment. During short-term follow up, one patient complained of new headaches, and three of recurrent hyperlacrimation of the irradiated eye, one with change of taste perception. None of the patients developed dysfunctions of the pituitary gland, neuropathy, retinopathy or brain necrosis. One patient complained of scotoma and visual disorder during FSRT, which receded completely after application of steroids. However, after termination of steroid medication, symptoms returned; the ONSM was removed surgically 6 months after completion of FSRT. In this patient, a dose of 52.2 Gy had been applied, in single doses of 1.8 Gy.

### Visual outcome

Prior to RT, 29 patients complained about any kind of visual impairment including visual field deficits, diplopia or amaurosis (see table 1). Of these patients, 15 had been treated with prior surgical resection (52%), and 14 patients were treated with FSRT as primary treatment without prior surgical intervention (48%). Therefore, of all patients treated with prior surgical resection, 15 out of 21 (71%) presented with pre-existing visual deficits, of which 4 presented with complete amaurosis. In the group without surgical intervention, 14 out of 19 (74%) complained of visual deficits, with 2 patients presenting with complete amaurosis.

**Figure 2:**
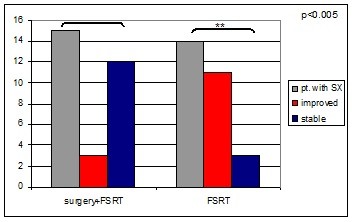
**Patients with visual impairment prior to FSRT and development of symptoms during follow up**. Patients previously treated with surgical interventions showed higher numbers of symptoms prior to RT. During follow-up, surgery-naive patients demonstrated significantly higher rates of improvements.

During follow-up after FSRT, vision improved in 12 out of 27 patients with pre-existing impairments (44%). In the surgery group, 3 patients showed improvement (20%), whereas in the RT-only group 9 patients showed symptom improvement during follow up (75%; Figure 2). This difference was statistically significant at p < 0.005.

Only one patient developed visual impairment after FSRT with respect to visual field deficits, and two patients complained of slight decrease of vision acuity. No severe side effects could be observed, and none of the patients developed new visual deficits.

**Figure 3:**
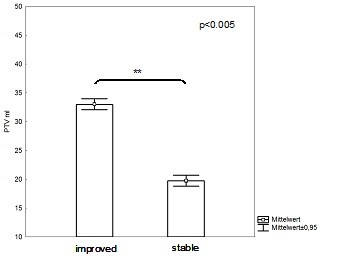
**Correlation of PTV-size and visual outcome**. Patients with smaller treatment volumes were more likely to present with stable disease, whereas patients with larger volumes showed a higher rate of visual improvement during follow-up.

With respect to treatment volume during FSRT, patients with larger target volumes showed significantly more improvement of visual function than patients with smaller volumes (p < 0.005; Figure 3).

## Discussion

The present manuscript supports previous reports and reinforces that high precision radiotherapy offers a highly effective treatment modality for patients with ONSM. Local control rates are excellent, and the rates of treatment-related side effects are minimal. The best response with respect to improvement of vision can be observed in patients treated with FSRT as primary definitive treatments, whereas surgically pre-treated patients demonstrate lower rates of improving symptoms. Additionally, patients with larger treatment volumes are more likely to show improvement of pre-exisiting clinical symptoms.

Several groups have reported their clinical results of precision radiotherapy for the treatment of ONSM [6,8-10,16,19-21]. All authors agree that local control can be achieved safely with very low recurrence rates as well as low rates of treatment-related side effects. However, since most patients with ONSM are younger patients with long-term survival after treatment, not only prevention of side effects if of high importance, but also improvement of pre-existing clinical symptoms.

A number of studies have shown that FSRT in addition to preservation of visual function, even improvement in pre-existing clinical symptoms, such as visual acuity, can be achieved [4,8,9,11,16,21,22]. At MGH in Boston, 25 patients with ONSM treated with highly conformal radiotherapy using protons or photons reported 95% improvement or stable vision during follow-up [22]. Turbin and colleagues published retrospective data on 64 patients with ONSM treated with observation, surgery, surgery combined with radiotherapy as well as radiation alone: Of all patients, the group treated with radiation alone showed the best results with respect to vision preservation, although about one third of the patients developed treatment-related side effects such as retinopathy or temporal lobe reactions [7]. However, no direct link to the implemented radiation techniques was reported, and due to the multicentric nature of the study it is most likely that not all centers treated with the most advanced methods available at that time. Therefore, direct comparison with modern stereotactic methods or proton radiotherapy might not be possible. With reports focussing on modern stereotactic techniques, improvement in visual function was reported to be between 42% and 85% [8,9,11,23,24].

Since in the past, neurosurgical resection was often considered the standard approach for ONSM, individual weighing of the risk-benefit-ratio in patients with ONSM is required, when deciding for a specific therapy. Until now, no direct randomized trials compare surgery with radiation. However, some authors have addressed side effects after both treatments: Andrewas and colleagues could show in 30 patients with ONSM that, compared to historical controls, patients treated with RT showed no evidence of tumor progression, and 150% higher probability of visual improvement during follow-up [9]. Turbin and colleagues evaluated 64 patients with ONSM, comparing observation, surgery, surgery and radiation as well as radiation alone. The results demonstrated that patients receiving radiation alone showed the highest rate of vision preservation [7]. In all studies, radiation doses between 50 and 55 Gy were applied. In our group a median dose fo 54 Gy in single fraction of median 1.8 Gy were applied, which is well in line with the reported doses in the literature.

Our data presented in the present manuscript also show that stable or improved vision can be achieved in the majority of patients with ONSM treated with FSRT. From the present data analyzed, we must emphasize that patients treated with radiation therapy as primary definitive treatment demonstrate the best improvement in visual deficits: Patients previously treated with surgical resection, although not showing a difference in local tumor control or toxicity, show lower rates of improvement with respect to visual deficits. This is most likely due to the differences in damage to the optic structures leading to visual impairment. Patients after surgery appear to present with more severe and irreversible symptoms, whereas patients in the radiation-only group suffer from symptoms caused by compression of the ONSM of normal tissue structures. In the orbial region very small changes in normal tissue and tumor geometry can have a major impact on organ and nerve function due to the very narrow architecture. Thus, imaging-defined stable tumors potentially associated with a slight tumor regression (not visible on imaging) may lead to a major decompression (due to the tight anatomical structures), improvement in visual impairment caused by tumor compression or more likely after FSRT.

This can be supported by the effect of tumor size on visual outcome: Additionally, our study revealed that patients with larger tumor volumes and thus larger treatment volumes for radiation demonstrate higher rates of improvement with respect to visual function, most likely since these symptoms originate from tumor mass effect declining after FSRT. However, in total, results are based on a limited number of patients, and no formal randomized clincial trial was performed to compare different treatment modalities.

## Conclusion

In conclusion, FSRT can achieve long-term control of ONSM without high-rates of treatment related side effects. Patients treated with prior surgical resection show reduced improvement of visual function, supporting the idea that surgery might be better reserved for large tumors with major compression and subsequent clinical symptoms, such as exophthalmia. For tumors with compression and acute symptomas surgery must be evaluated. Doses of 50-55 Gy in normofractionated regimens show safety even after long-term follow-up. Novel concepts evaluating hypofractionated regimens are currently under investigation, but should be applied cauteously within clinical trials to safely assess toxicity.

## Conflict of interest

The authors declare that they have no competing interests.

## Authors' contributions

SC, JD and SR treated the patients. SA, SR and SC collected the data. SC and SA evaluated the dataset and performed statistical analysis. SC, SA, SR, TW and JD wrote and edited the manuscript. All authors read and approved the manuscript.
